# Changes in maternal blood glucose and lipid concentrations during pregnancy differ by maternal body mass index and are related to birthweight: A prospective, longitudinal study of healthy pregnancies

**DOI:** 10.1371/journal.pone.0232749

**Published:** 2020-06-23

**Authors:** Marie Cecilie Paasche Roland, Tove Lekva, Kristin Godang, Jens Bollerslev, Tore Henriksen

**Affiliations:** 1 Department of Obstetrics, Oslo University Hospital, Oslo, Norway; 2 National Advisory Unit on Women’s Health, Oslo University Hospital, Oslo, Norway; 3 Research Institute of Internal Medicine, Oslo University Hospital, Oslo, Norway; 4 Department of Endocrinology, Oslo University Hospital, Oslo, Norway; 5 Department of Clinical Medicine, University of Oslo, Oslo, Norway; University of Hawai’i at Manoa College of Tropical Agriculture and Human Resources, UNITED STATES

## Abstract

**Background:**

Maternal obesity is increasing worldwide but the consequences for maternal physiology and fetal growth are not fully understood.

**Objective:**

To study whether changes in glucose and lipid metabolism during pregnancy differ between women with normal weight and overweight/obesity, and investigate which of these metabolic factors are associated with birthweight.

**Design:**

Prospective, longitudinal study.

**Setting:**

Department of Obstetrics, Oslo University Hospital, Rikshospitalet.

**Population:**

1031 healthy pregnant women with singleton pregnancies.

**Methods:**

Blood samples from early and late pregnancy were analyzed for fasting glucose, insulin and lipids (total cholesterol, HDL-cholesterol, LDL-cholesterol and triglycerides). Associations between metabolic factors and birthweight (z-scores) were explored by linear regression models. Main Outcome Measures: Group-dependent longitudinal changes in glucose and lipids and their association with birthweight (z-scores).

**Results:**

Compared to women with normal weight (BMI < 25), women with overweight (BMI 25–29.9) and obesity (BMI > 30) had significantly higher fasting glucose (4.54, 4.68 and 4.84 mmol/l), insulin (23, 33 and 50 pmol/l), total cholesterol (4.85, 4.99 and 5.14 mmol/l), LDL-C (2.49, 2.66 and 2.88 mmol/l) and triglycerides (1.10, 1.28 and 1.57 mmol/l), but lower HDL-C (1.86, 1.75 and 1.55 mmol/l). BMI (B 0.05, 95% CI 0.03–0.06, p<0.001), gestational weight gain (GWG) (B 0.06, 0.05–0.08, p<0.001) and an increase in fasting glucose (B 0.30, 0.16–0.43, p<0.001) were positively associated with birthweight, whereas a decrease in HDL-C (B -0.72, -0.96- -0.53, p<0.001) had a negative association with birthweight.

**Conclusions:**

Overweight/obesity was associated with an unfavorable metabolic profile in early pregnancy which was associated with increased birthweight. However, modifiable factors like gestational weight gain and an increase in fasting glucose were identified and can be targeted for interventions.

## Introduction

The intrauterine environment in which a fetus develops has increasingly been recognized as a determinant of not only pregnancy outcome at birth, but of future health [1]. Pregnancy is characterized by physiological adaptations in weight, glucose and lipid metabolism to ensure that the increasing nutritional demands of the mother, the placenta and the fetus are met. Gestational weight gain (GWG) includes growth of the fetus, placenta and uterus, increase in maternal plasma volume and accumulation of maternal fat mass, adding up to an average of 12.5 kg [2]. As the human fetus is highly dependent on glucose derived from the maternal circulation for growth and development, glucose homeostasis is maintained by increased hepatic glucose production, reduced insulin sensitivity and consequently increased insulin production [3, 4]. The lipid metabolism in pregnancy is characterized by a marked hyperlipidemia, in which particularly triglycerides increase and cholesterol levels rise to a lesser degree [5].

Adverse pregnancy outcomes can result when weight, gestational weight gain and changes in maternal metabolism exceed physiological levels needed to obtain a successful pregnancy outcome. High maternal body mass index (BMI), commonly used as a proxy for excess body fat, is consistently associated with increased risk of most pregnancy complications and long term outcomes for mother and child [5, 6]. The Hyperglycemia and Adverse Pregnancy Outcome Study (HAPO) showed that hyperglycemia in pregnancy is independently and linearly associated with risk of adverse pregnancy outcomes for mother and child [7]. A similar association was shown for maternal BMI [8]. We have previously shown how maternal BMI influences fetal growth measured as birthweight, intrauterine growth and percentage fat estimated by DXA in newborns, both directly as well as through increasing placental weight [9–13].

Maternal BMI increases in most populations globally and obese pregnant women have increased risk of many pregnancy complications, in particular gestational diabetes (GDM), large for gestational age offspring (LGA) and the need for cesarean section which are increased two- or threefold [5]. The combination of obesity and hyperglycemia represents a particular challenge in obstetrics [14, 15]. Excess fetal growth is accompanied by increased risk of complications at birth, exemplified by shoulder dystocia, and is increasingly recognized as a risk factor for childhood obesity [14]. The majority of LGA infants are born to obese mothers without GDM and it is therefore of great interest to understand which biological effects besides hyperglycemia that exert these effects [15]. The role of hyperlipidemia in fetal growth is less studied than hyperglycemia in normal pregnancy [16, 17] although triglycerides have been shown to be associated to birthweight in gestational diabetes mellitus (GDM) [18, 19].

Physiological metabolic changes occurring in glucose and lipid metabolism throughout pregnancy are affected by increased maternal BMI. However, how exaggerated or attenuated physiological changes due to increased maternal BMI affect fetal growth is not fully understood [14]. Further, whether fetal growth is influenced by unfavorable metabolic status as the woman enters pregnancy or by the resulting changes occurring during pregnancy needs clarification. These are knowledge gaps to be filled in order to understand which women are at increased risk for abnormal fetal growth and secondly to choose the correct timing and type of intervention to reduce their risk of adverse outcomes on both short and long term.

We hypothesized that both the metabolic status in early pregnancy as well as the longitudinal changes in glucose and lipid metabolism differ between women categorized according to BMI and that these changes influence fetal growth.

## Materials and methods

### Population and data collection

The STORK study included 1031 healthy pregnant women who gave birth at Oslo University Hospital Rikshospitalet between 2002 and 2008. Inclusion criteria were singleton pregnancies in healthy women of Scandinavian heritage. Exclusion criteria were multiple pregnancies, pre-existing diabetes mellitus, fetal malformations discovered at routine ultrasound examination and major maternal comorbidities. Details on the recruitment and flowchart of inclusion of participants have been published [9]. Briefly, each woman had four antenatal visits, scheduled at 14–16 (v1), 20–22 (v2), 30–32 (v3) and 36–38 (v4) weeks of pregnancy. Data on age, parity, obstetric history, educational level and smoking status were registered. Parity was coded as P0 for primigravida and P1 for one or more previous births. Gestational age was based on ultrasound biometric measures made at weeks 17–19. BMI (kg/m^2^) was calculated by height and weight. Maternal height was measured at the first visit and weight was measured by a calibrated scale at each visit. Gestational weight gain was calculated as the difference between weights measured at visit 4 and visit 1. Measured weight at the first visit was used instead of pre pregnancy weight to avoid false self-reporting of pre pregnancy weight. Women were categorized according to BMI using World Health Organization (WHO) categories into three groups, normal weight BMI < 25.0, overweight BMI 25.0–29.9 and obesity BMI >30.0 [2]. Outcomes of pregnancy were collected from hospital records. Birthweight was measured by a calibrated scale. Birthweight is given as birthweight for gestational age and sex-specific z-scores [16].

### Analyses of blood samples

Blood samples were collected at 14–16 weeks (v1) and 36–38 weeks (v4) and drawn in the morning between 07:30 and 08:30 after an overnight fast, centrifuged and stored at -80 °C. Fasting glucose was measured in serum samples, using the hexokinase method at the accredited laboratory at Oslo University Hospital Aker (Hitachi Modular P800 with reagents from Roche). Insulin levels were assayed in duplicate by RIA (Diagnostic Products Corp, Los Angeles, CA, USA) as previously reported [17]. Lipids (total cholesterol, HDL-C and triglycerides) were measured at the accredited laboratory at the Department for Medical Biochemistry, Oslo University Hospital, Rikshospitalet. All samples from a given patient were analyzed at the same time to minimize the run-to-run variability. Intra- and interassay coefficients of variation were less than 5% for all. Low-density lipoprotein cholesterol (LDL-C) was determined by Friedewald’s formula [18].

### Statistics

Descriptive statistics were used to characterize the population. Data are expressed as mean ± SD when normally distributed and median (25th, 75th percentile) when skewed. Bivariate associations were explored by scatter plots and correlation analyses. Longitudinal changes between v1 and v4 in the same individual were compared by paired t-tests or Wilcoxon Signed Rank Test as appropriate. Differences between groups according to BMI-categories were compared by oneway ANOVA (corrected for multiple testing by Bonferonni posthoc test) or Kruskal Wallis test.

Associations between maternal characteristics and the outcome variable birthweight (given as birthweight for gestational age and sex-specific z-scores) were analyzed by univariate and multiple linear regression models. Four multiple models were explored. Model 1 included metabolic factors measured in early pregnancy (v1), model 2 metabolic factors measured in late pregnancy (v4) and model 3 included the changes in variables between measurements made between early and late pregnancy (v4-v1). Model 4 is a multiple regression model including variables from model 1 and 3. Variables with a p-value < 0.1 in univariate analyses were considered in the multiple regression models. A p-value < 0.05 was considered statistically significant. All analyses were done by SPSS 23 (IBM Corp).

### Ethical statement

Written informed consent was obtained from all participants in the study. All clinical investigations were conducted according to the principles expressed in the Declaration of Helsinki. The study was approved by the Regional Committee for Medical Research Ethics, Southern Norway, Oslo, Norway (S-01191).

## Results

The maternal and neonatal characteristics of the cohort are presented in Table 1.

**Table 1:** Characteristics of mothers and neonates in the total cohort and in groups according to BMI.

Maternal variables	Total cohort N = 1031			BMI<25 N = 628			BMI 25–29.9 N = 286			BMI>30 N = 89			Difference between groups
	Mean	SD	%	Mean	SD	%	Mean	SD		Mean	SD	%	
Age (yrs)	31.3	3.9		31.1	3.8		31.5	3.9		31.5	4.4		ns
P0			53			55			52			46	ns
Smoking			2.7			2.2			3.1			5.6	ns
Education > 12 yrs			86.6			89.5			85.0			65.2	#
Married			98			98			97.5			99	ns
BMI v1	24.5	3.9		22.2	1.7		26.8	1.3		33.5	3.2		#
GWG v4-v1 (kg)	10.6	3.5		10.6	3.2		10.6	3.7		9.8	4.7		ns
GDM			12.5			9.3			16.4			21.8	#
Preeclampsia			3.8			2.1			5.9			9.0	#
**Neonatal variables**													
Birthweight (g)	3588	574		3510	548		3706	577		3757	644		#
Birthweight (z-score)	0.13	1.02		-0.05	0.97		0.40	1.04		0.51	1.2		#
Gestational age				39.4	1.8		39.5	1.9		39.5	1.9		ns
SGA < 10 p			7.6			9.4			3.5			7.9	#
LGA > 10 p			14			9.7			20.7			22.5	#
Fetal sex (% boys)			52.6			54.0			54.0			56.2	ns
Placental weight (g)	711	156		692	153		736	148		768	644		#

^#^p<0.05, differences between groups, One-way ANOVA

Approximately 61% had a BMI < 25 in early pregnancy. Level of education was negatively associated with increasing maternal BMI. Overweight and obese women experienced higher rates of complications in pregnancy like gestational diabetes mellitus (GDM) and preeclampsia (PE) compared to normal weight women. Measures of fetal growth were higher, both measured as birthweight (grams) and proportion of large for gestational age newborns (LGA).

**Fig 1:**
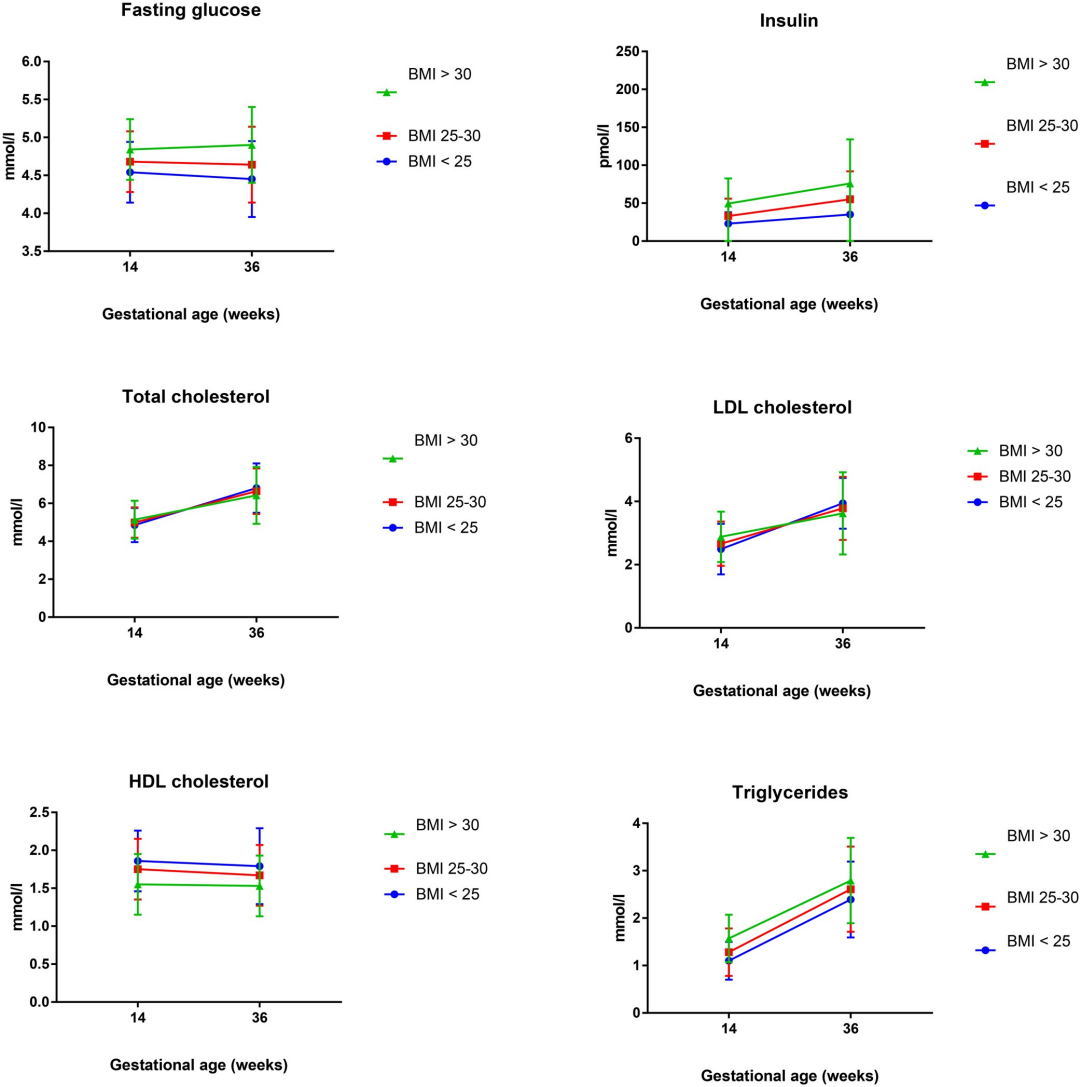
Longitudinal changes in fasting glucose, insulin and lipids according to groups of BMI.

The results of fasting glucose, insulin and lipids measured in early and late pregnancy are presented in Table 2 and Fig 1.

**Table 2:** Glucose, insulin and lipids throughout pregnancy in the total cohort and in groups according to BMI.

	Total cohort n = 1031		BMI<25 n = 628		BMI 25–29.9 = 286		BMI>30 = 89		Difference between groups
	Mean	SD	Mean	SD	Mean	SD	Mean	SD	
Fasting glucose v1	4.61	0.4	4.54	0.4	4.68	0.4	4.84	0.4	#
Fasting glucose v4	4.54	0.5	4.45	0.5	4.64	0.5	4.90	0.5	#
Δ Fasting glucose v4-v1	-0.06*	0.5	-0.09*	0.5	-0.04	0.4	0.07	0.5	#
Insulin v1 (median/IQR)	27	18–40	23	15–33	33	23–46	49.5	33–75	«»
Insulin v4 (median/IQR)	42	28–65	35	24–50	55	37–80	76	58–124	«»
Δ Insulin v4-v1	15¤		14¤		25¤		26.5¤		«»
Total chol v1	4.92	0.9	4.85	0.9	4.99	0.8	5.14	1.0	#
Tot chol v4	6.71	1.3	6.80	1.3	6.64	1.2	6.42	1.5	#
Δ Tot chol v4-v1	1.8*	1.0	1.95*	1.0	1.66*	1.0	1.28*	1.0	#
HDL chol v1	1.8	0.4	1.86	0.4	1.75	0.4	1.55	0.4	#
HDL chol v4	1.72	0.4	1.79	0.5	1.67	0.4	1.53	0.4	#
Δ HDL chol v4-vl	-0.07*	0.4	-0.07*	0.3	-0.08*	0.4	-0.006	0.3	ns
LDL chol v1	2.58	0.8	2.49	0.8	2.66	0.7	2.88	0.8	#
LDL chol v4	3.87	1.2	3.94	1.2	3.78	1.0	3.62	1.3	#
Δ LDL chol V4-v1	1.29*	0.9	1.44*	0.9	1.13*	0.9	0.73*	0.8	#
Triglycerides v1	1.19	0.7	1.10	0.4	1.28	0.5	1.57	0.5	#
Triglycerides v4	2.48	0.8	2.39	0.8	2.61	0.9	2.79	0.9	#
Δ Triglycer v4-v1	1.3*	0.7	1.29*	0.6	1.35*	0.7	1.23*	0.8	ns

^#^ p<0.05 One-way ANOVA, comparing differences between groups

^«»^p<0.001 Kruskal Wallis test, comparing differences between groups, non-parametric test

* p<0.05 paired t-test, comparing longitudinal changes in each individual

^¤^ p<0.01Wilcoxon Signed Rank Test, comparing longitudinal changes in each individual, non-parametric test

At the first visit at week 14–16 there were significant differences between the groups, demonstrating higher concentrations of fasting glucose, insulin and all lipids in the overweight and obese groups compared to the normal weight group, with the exception of HDL-C which was higher in lean women.

Significant longitudinal changes were observed in glucose, insulin and all lipids for the total cohort. Fasting glucose decreased throughout pregnancy in the total cohort, but this decrease was statistically significant only in lean women and obese women had a tendency to increase their fasting glucose by late pregnancy. Insulin concentrations were higher at the end of pregnancy for all groups and the increase was higher in the overweight and obese groups.

Total cholesterol and LDL-C increased significantly throughout pregnancy, but there was a less pronounced rise in the overweight and obese groups. Triglycerides had the largest increase during pregnancy, but no group-dependent differences were observed. HDL-C displayed a slight decrease from early to late pregnancy which was statistically significant, except in the obese group, but no group-dependent differences.

**Fig 2:**
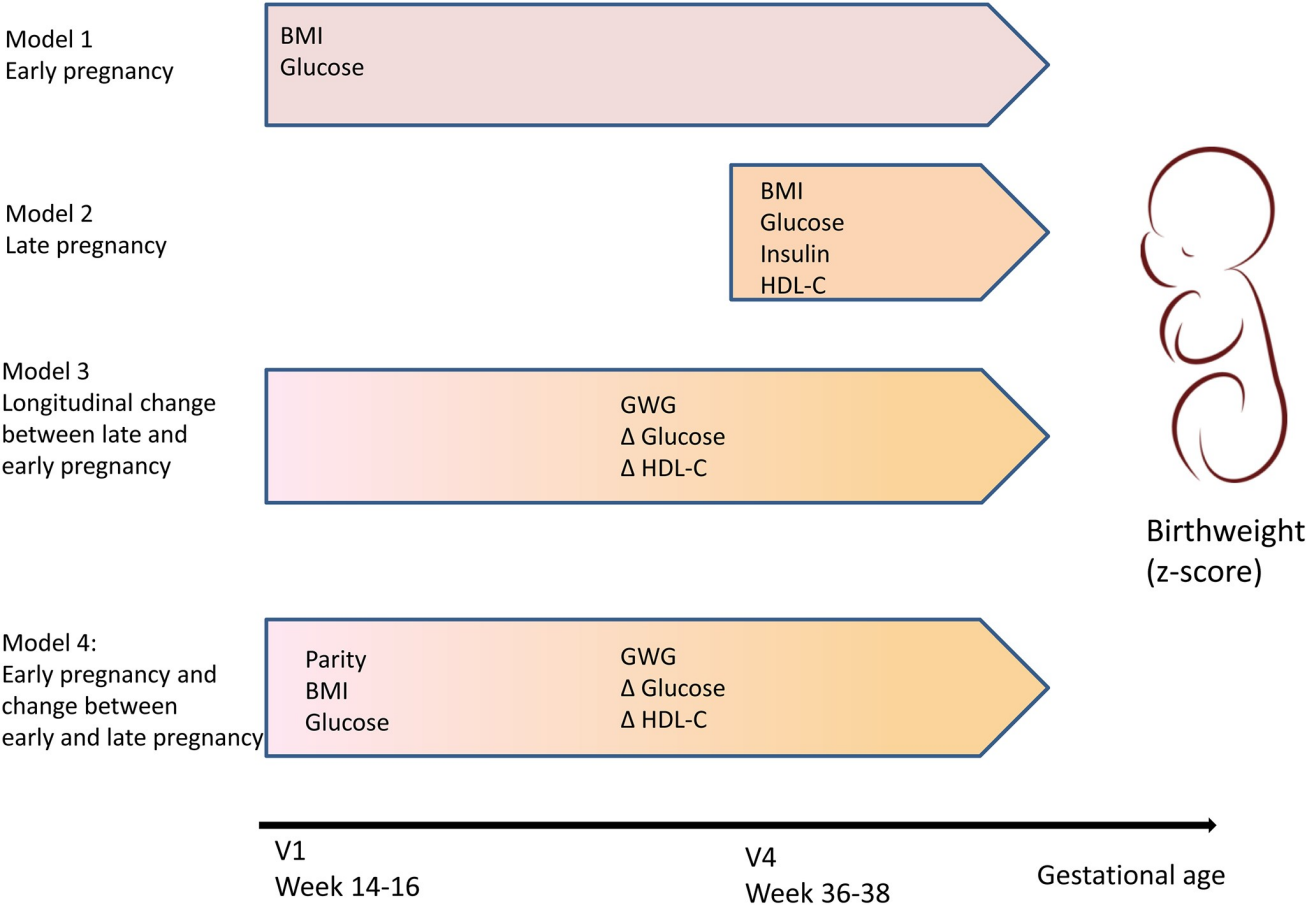
Figure illustrating the results from multiple regression analyses presenting the maternal variables associated with birthweight measured in early pregnancy (model 1), late pregnancy (model 2), the longitudinal changes between late and early pregnancy (model 3) and the final model including both measurements from early pregnancy and the longitudinal changes (model 4).

The results of the multiple regression models exploring the associations between maternal metabolic factors and birthweight are presented in Table 3 and illustrated in Fig 2.

**Table 3:** Relations between glucose, insulin and lipid concentrations in early pregnancy (v1) and late pregnancy (v4) and longitudinal changes (v4-v1) and birthweight (z-score). Results from univariate and multiple linear regression models.

Variables	Univariate B (95% CI)	p-value	Model 1 (v1) B (95% CI)	p-value	Model 2 (v4) B (95% CI)	p-value	Model 3 (v4-v1) B (95% CI)	p-value	Model 4 (Model 1 and 3) B (95% CI)	p-value
Parity	0.49 (0.37–0.62)	<0.001							0.44 (0.32–0.56)	<0.001
BMI v1	0.06 (0.05–0.08)	<0.001	0.05 (0.03–0.07)	<0.001					0.05 (0.03–0.06)	<0.001
BMI v4	0.07 (0.05–0.08)	<0.001			0.06 (0.04–0.08)	<0.001				
Gestational weight gain	0.05 (0.03–0.07)	<0.001					0.05 (0.03–0.07)	<0.001	0.06 (0.04–0.08)	<0.001
Glucose v1	0.49 (0.32–0.66)	<0.001	0.33 (0.15–0.52)	<0.001					0.49 (0.31–0.67)	<0,001
Glucose v4	0.51 (0.39–0.64)	<0.001			0.44 (0.30–0.57)	<0.001				
Δ glucose v4-v1	0.29 (0.15–0.43)	<0.001					0.30 (0.15–0.45)	<0.001	0.30 (0.16–0.43)	<0.001
Insulin v1	0.005 (0.002–0.007)	<0.001	0.001 (-0.003–0.003)	0.9						
Insulin v4	0.002 (0.000–0.003)	<0.01			-0.002 (-0.004--0.001)	<0.01				
Δ insulin v4-v1	0.001 (-0.001–0.002)	0.4					-0.001 (-0.003–0.000)	0.2		
Chol tot v1	0.035 (-0.04–0.11)	0.4								
Chol tot v4	-0.024 (-0.08–0.03)	0.4								
Δ chol tot v4-v1	-0.041 (-0.11–0.03)	0.2								
LDL-C v1	0.083 (-0.002–0.17)	0.056	0.028 (-0.006–0.12)	0.5						
LDL-C v4	0.022 (-0.03–0.08)	0.4								
Δ LDL-C v4-v1	0.008 (-0.07–0.08)	0.8					0.07 (-0.07–0.76)	0.9		
HDL-C v1	-0.23 (-0.39--0.08)	<0.005	-0.074 (-0.23–0.14)	0.4						
HDL-C v4	-0.51 (-0.66--0.37)	<0.001			-0.44 (0.59--0.29)	<0.001				
Δ HDL-C v4- v1	-0.64 (-0.08--0.44)	<0.001					-0.68 (-0.89--0.47)	<0.001	-0.72 (-0.90--0.53)	<0.001
TG v1	0.21 (0.06–0.36)	<0.01	-0.05 (-0.23–0.14)	0.6						
TG v4	0.10 (0.02–0.18)	<0.05			-0.03 (-0.11–0.05)	0.4				
ΔTG v4-v1	0.10 (0.002–0.20)	**<0.05**					**-0.02 (-0.12–0.09)**	**0.9**		

Model 1: metabolic factors measured at v1

Model 2: metabolic factors measured at v4

Model 3: changes in metabolic factors between early and late pregnancy (v4-v1)

Model 4: metabolic factors at v1 and changes in metabolic factors v4-v1

In univariate analyses metabolic variables associated with birthweight included BMI, GWG, fasting glucose and insulin as well as triglycerides and HDL-C, whereas total cholesterol and LDL-C were not. Model 1 and Model 2 confirmed that BMI and fasting glucose were associated with birthweight regardless of whether they were measured in early or late pregnancy. In Model 3 we found that GWG and an increase in glucose were positively associated with birthweight, whereas a decrease in HDL-C was negatively associated with birthweight. Finally, in model 4 we showed that the metabolic profile in early pregnancy measured as BMI, fasting glucose and HDL-C, as well as the changes in these parameters during pregnancy had independent effects on birthweight, even when adjusted for parity.

## Discussion

We have demonstrated how longitudinal changes in glucose, insulin and lipids throughout pregnancy in a large cohort of healthy pregnant women differ between normal weight and overweight and obese women. Overweight and obese women had higher fasting plasma glucose, insulin and all fractions of lipids, except HDL-C, compared to normal weight women. However, although all lipids (except HDL-C) increased during pregnancy, the magnitude of change in lipids was either less prominent in overweight and obese women (as seen in total cholesterol and LDL-C) or similar between the groups (as seen in HDL-C and triglycerides).

We confirmed positive effects of BMI, GWG and fasting glucose on birthweight and further identified a negative effect of HDL-C on birthweight. These effects were identified already from early pregnancy. In addition the changes occurring in glucose and HDL-C between early and late pregnancy had independent effects.

Our findings that overweight and obese women demonstrate a more atherogenic lipid profile in early pregnancy when compared to normal weight women are in line with results from several other groups [19–22]. The longitudinal increase in total cholesterol, LDL-C and triglycerides are comparable to data published by Farias et al in which total cholesterol, LDL-C and triglycerides increased linearly with gestation, whereas HDL-C peaked in early third trimester and declined towards term [21]. We found that in overweight and obese pregnant women the magnitude of longitudinal changes in lipids were less pronounced than in normal weight women, consistent with findings by Bozkurt et al in a cohort of 220 women.

The associations between hyperglycemia and macrosomia/LGA are well known and have been shown in large international studies like HAPO, as well as demonstrated previously in our cohort [9, 15]. We found that fasting glucose is consistently associated with increased birthweight, both when measured in early and late pregnancy. Most studies have demonstrated that hyperglycemia in third trimester is associated with increased birthweight, but moreover, we here demonstrate that the positive association was found as early as in week 14. In lean women a slight decrease in fasting glucose towards term was observed, as previously published by Catalano et al [3, 4]. In contrast, we found that overweight and obese women maintained or increased their fasting glucose towards term, and this change was also a factor associated with increased birthweight. While hyperglycemia and BMI have independent effects on birthweight [15], the combination of the two has greater effect than each alone [8, 23].

In normal pregnancy basal endogenous glucose production increases by approximately 30% by the end of gestation accompanied by a substantial increase in fasting insulin levels. This results in a decrease in circulating fasting glucose towards term [4]. This is thought to be the result of an increase in plasma volume as well as increased glucose utilization by the feto-placental unit. Peripheral insulin sensitivity is decreased by approximately 50% by late gestation [3]. In women with GDM suppressed endogenous glucose production and more pronounced decrease in peripheral insulin sensitivity contribute to fasting hyperglycemia [24]. Previous results from our cohort have demonstrated deterioration of β-cell function adjusted for insulin sensitivity in both glucose-tolerant and glucose-intolerant women. This failure to compensate for decreased insulin sensitivity was accentuated in overweight women [17]. The mechanisms linking obesity to impaired glucose metabolism in pregnancy is only partly understood. Studies on the insulin-signaling cascade have revealed 25% lower glucose uptake in biopsied skeletal muscle from women with GDM due to lower contents of one of the signaling molecules, insulin receptor substrate 1 (IRS1)[25]. A link between obesity and impairment of the insulin-signaling pathway was demonstrated in a study where of mice given a high-fat diet during pregnancy resulted in offspring with both increment in body mass and adiposity also had lower levels of IRS1 [26].

Increased BMI in early pregnancy was accompanied by alterations in lipid profile. The role of other BMI-related metabolic factors like lipids in fetal growth has been more extensively studied in diabetic pregnancies than in healthy pregnancies. The role of individual lipid fractions in normal pregnancies has not been fully established. In well-controlled diabetic pregnancies maternal triglycerides have been linked to fetal growth, in particular neonatal fat mass and LGA [27]. The correlation between triglycerides and birthweight seen in our cohort of healthy women was independent of fasting glucose, contradicting the proposed notion that maternal lipids are predictors of fetal growth only in GDM pregnancies. However, the effect of triglycerides on birthweight disappeared when adjusting for maternal BMI, bringing up the question whether the conflicting results previously reported between GDM and non-GDM could possibly be partly explained by factors related to adiposity in addition to hyperglycemia. The strongest correlation between maternal lipids and birthweight in our material was the negative association between HDL-C in early pregnancy and birthweight, which remained statistically significant even after adjusting for BMI and GWG. This is in line with previous reports, although HDL-C was measured later in pregnancy [28].[5] HDL-C has been shown to increase with gestational age, peak in the third trimester and decline towards term [20, 21]. We only measured HDL-C in early pregnancy and late third trimester and hence could not show the peak in the early third trimester. Nevertheless, the slight fall in HDL-C from early to late pregnancy in the total cohort was negatively associated with birthweight. The positive effect of GWG on birthweight was independent of BMI and metabolic status in early pregnancy, as well as changes occurring in both glucose and lipids throughout pregnancy. GWG therefore represents a factor that can be modified in cases of women entering pregnancy with an unfavorable status in terms of their weight or metabolism.

This study has a large sample size with more than 1000 women that are well characterized with detailed data on glucose, insulin and lipids as well as maternal and neonatal outcomes. The longitudinal design with blood samples drawn both in early pregnancy and third trimester allowed us to evaluate how metabolic factors at different gestational ages as well as the longitudinal changes in these factors relate to fetal growth. Further, the combination of glucose and lipid measurements and clinical data like GWG enabled us to perform multiple regression analyses, identifying variables with independent effects. As the cohort consisted of healthy women our study sheds light on the physiology and fetal growth of normal pregnancy rather than on pathophysiology related to specific complications of pregnancy.

## Conclusion

Taken together our findings show that overweight and obesity in early pregnancy were associated with an unfavorable metabolic profile which increased with increasing BMI. High BMI and accompanying disturbances in glucose and lipid metabolism found in early pregnancy were associated with increased birthweight. The benefits of entering pregnancy with normal BMI and metabolic status should therefore ideally be communicated to women prior to pregnancy. However, factors like gestational weight gain and an increase in fasting glucose are additional and modifiable factors that can be targeted for interventions, which should start as early as possible in pregnancy. Further studies should focus on identifying other BMI-related factors that exert biological effects and hence contribute to the risk of adverse pregnancy outcomes associated with high BMI. The current study pinpoints HDL-C as an independent BMI-related factor related to fetal growth.
